# Preparation of Electrospun Gelatin Mat with Incorporated Zinc Oxide/Graphene Oxide and Its Antibacterial Activity

**DOI:** 10.3390/molecules25051043

**Published:** 2020-02-26

**Authors:** Honghai Li, Yu Chen, Weipeng Lu, Yisheng Xu, Yanchuan Guo, Geng Yang

**Affiliations:** 1 Key Laboratory of Photochemical Conversion and Optoelectronic Material, Technical Institute of Physics and Chemistry, Chinese Academy of Sciences, Beijing 100190, China; 15061881525@163.com (H.L.); chenyubrc@mail.ipc.ac.cn (Y.C.); yanggeng0131@163.com (G.Y.); 2 School of Chemical Engineering, East China University of Science and Technology, Shanghai 200237, China; 3 School of Future Technology, University of Chinese Academy of Sciences, Beijing 100049, China

**Keywords:** gelatin fibers, electrospinning, ZnO/GO, antibacterial activity, wound dressing

## Abstract

Current wound dressings have poor antimicrobial activities and are difficult to degrade. Therefore, biodegradable and antibacterial dressings are urgently needed. In this article, we used the hydrothermal method and side-by-side electrospinning technology to prepare a gelatin mat with incorporated zinc oxide/graphene oxide (ZnO/GO) nanocomposites. The resultant fibers were characterized by field emission environment scanning electron microscopy (FESEM), energy-dispersive X-ray spectroscopy (EDX), X-ray diffractometry (XRD) and Fourier transform infrared spectroscopy (FTIR). Results indicated that the gelatin fibers had good morphology, and ZnO/GO nanocomposites were uniformly dispersed on the fibers. The loss of *Escherichia coli* (*E. coli*) and *Staphylococcus aureus* (*S. aureus*) viability were observed to more than 90% with the incorporation of ZnO/GO. The degradation process showed that the composite fibers completely degraded within 7 days and had good controllable degradation characteristics. This study demonstrated the potential applicability of ZnO/GO-gelatin mats with excellent antibacterial properties as wound dressing material.

## 1. Introduction

Almost all wounds are polluted by bacteria to varying degrees from the air or tissues near the wounds. How to effectively provide a sterile environment for wounds has always been a problem worth exploring in the field of wound dressing research [1,2]. Traditional wound dressings are mainly made of cotton and yarn; the material′s lack of antibacterial activity requires the need for it to be replaced frequently to keep the wound clean, which may cause secondary injury to the wound [3,4]. In addition, replacing wound dressings frequently not only generates medical waste, causing a lot of pollution to the environment and human beings but also wastes resources in subsequent treatment processes [5,6]. Therefore, a wound dressing made of biocompatible materials with good antibacterial activities and biodegradability has become a research hotspot.

Gelatin, hydrolyzed from collagen, is a kind of biopolymer protein. Good biocompatibility, biodegradability and low immunogenicity make it widely used as a raw material of medical materials [7,8], especially gelatin nanofibers based on electrospinning technology that have the features of large porosity, large specific surface area and strong adsorption. There are many small secondary structures on the surface of gelatin nanofibers similar to the structure of extracellular matrix (ECM). These properties enable gelatin nanofibers to promote the adhesion, migration, differentiation and growth of cells obviously [7,9] and have unique advantages in the field of medical dressings. In addition, zinc oxide (ZnO) nanoparticles with a diameter of 1–100 nm are a type of multifunctional fine inorganic material [10,11], which has the inherent advantages of antibacterial activities against bacteria [12,13] and are regarded as materials with biosafety and biocompatibility [14,15]; thus, it has spurred significant interest as an inorganic antibacterial agent. Recently, the encapsulation of ZnO nanoparticles into gelatin nanofibers is a promising approach to enhance the antimicrobial activity of nanoparticles due to improved stability and sustained release. Zhang et al. prepared gelatin/ethyl cellulose/ZnO nanofibers by electrospinning, promoting the dispersion of nanoparticles and improving the antibacterial activity against *S. aureus* (43.7–62.5%) [16]. Goyal et al. fabricated gelatin nanofibers loaded with cephalosporin and ZnO; the antibacterial assay showed that the minimum inhibitory concentration was 1.9 ± 0.2 μg mL^−1^ [17]. Although these works have improved the antibacterial activities of nanofibers, there are still two obstacles in combining ZnO nanoparticles and gelatin nanofibers: (1) the easy aggregation and settlement of ZnO nanoparticles hamper the incorporation of ZnO into gelatin nanofibers, (2) the adhesion between ZnO nanoparticles and gelatin is weak, which makes ZnO easy to fall off.

**Scheme 1:**
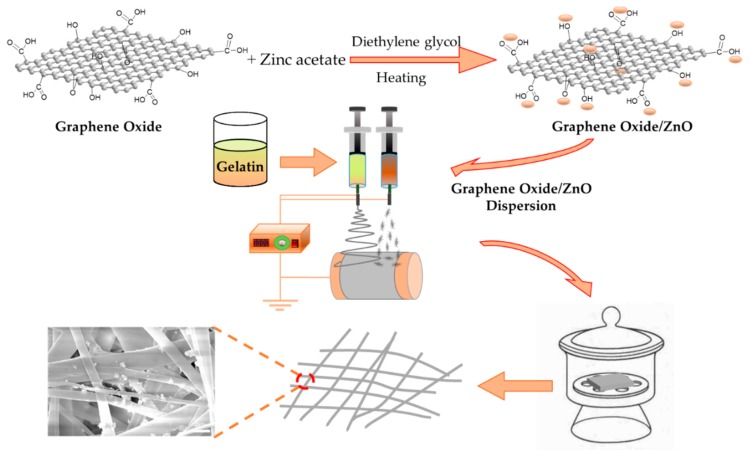
The preparation processes of ZnO/GO-gelatin fibers.

To solve the above problems, herein, we used graphene oxide (GO) as a substrate, enabling in situ formation of ZnO nanoparticles on GO uniformly in a hydrothermal environment. The hydrophilicity of GO can improve the stability of ZnO/GO in absolute ethanol and achieve the stable spray of ZnO/GO nanocomposites following the solvent. Utilizing side-by-side spray nozzle electrospinning, ZnO/GO nanocomposites were uniformly dispersed on the gelatin fibers as it formed (Scheme 1). Furthermore, GO sheets, covered mostly with epoxy, hydroxyl, carbonyl and carboxyl groups, can solve the problem that nanoparticles easily fall off from the surface of fibers by forming a stable interaction with the gelatin. Additionally, GO has also been reported for its significant antibacterial properties [18], which also enhances the antibacterial property of the resultant fibers. It is reasonable to believe that the ZnO/GO-gelatin fibers hold great potential to serve as a wound dressing.

## 2. Results and Discussion

### 2.1. Characterization of ZnO/GO-Gelatin Fibers

#### 2.1.1. Characterization of ZnO/GO Nanocomposites

**Figure 1:**
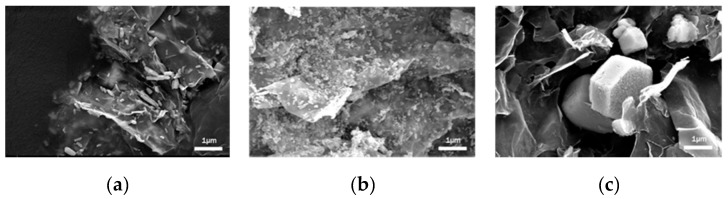
FESEM micrographs of (**a**) ZnO/GO-5 wt%, (**b**) ZnO/GO-10 wt%, (**c**) ZnO/GO-15 wt%.

In the hydrothermal reaction, zinc ion from zinc acetate bound to the oxygen atoms of negatively charged oxygen-containing functional groups on GO by electrostatic force and coordination reaction [19], the crystal nuclei grew up gradually, and finally, the ZnO/GO nanocomposites formed. Field emission environment scanning electron microscope (FESEM) micrographs (Figure 1a,b) indicate that regular rod-shaped ZnO nanoparticles were obtained with low GO concentration (5 wt%, 10 wt%). ZnO nanoparticles in ZnO/GO-10 wt% exhibited a smaller average diameter (47.4 nm, 50 samples collected) than that in ZnO/GO-5 wt% (77.9 nm). In general, the smaller size is beneficial to the antibacterial properties [20] and is more conducive for dispersion in ethanol solution. However, with 15 wt% GO added, the morphology of ZnO nanoparticles turned into hexagonal crystal, whose diameter was much larger than the ZnO with a low GO concentration (Figure 1c). In the process of electrospinning, oversized ZnO/GO nanocomposites lead to settlement in ethanol dispersion, which is difficult to spray following the ethanol; this is consistent with the phenomenon observed in the experiment. Therefore, ZnO/GO-10 wt% was chosen as the material to fabricate ZnO/GO-gelatin fibers and carry out the following characterization and experiments.

**Figure 2:**
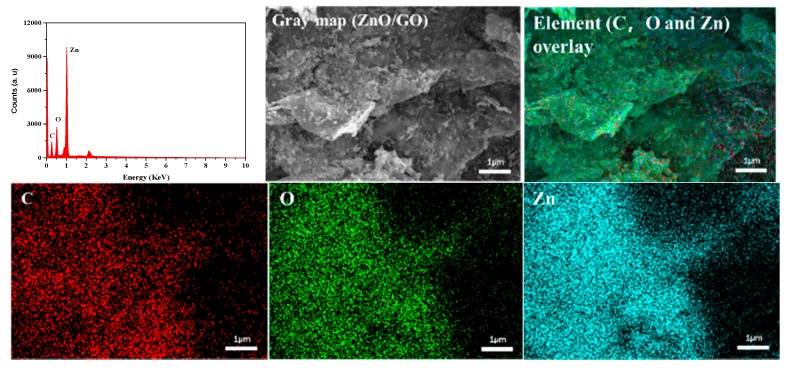
X-ray spectroscopy (EDX) analysis of ZnO/GO.

**Figure 3:**
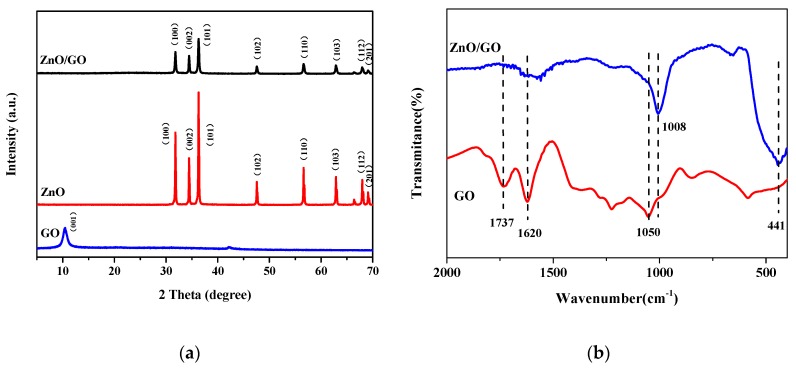
(**a**) XRD spectra of GO, pure ZnO and ZnO/GO, (**b**) Fourier transform infrared spectroscopy (FTIR) spectra of GO and ZnO/GO.

To study the structure of ZnO/GO, energy dispersive X-ray spectroscopy (EDX) analysis was carried out. As shown in Figure 2, there were three elements in ZnO/GO: C, O and Zn, element C represents the GO sheets, and the GO surface was covered by ZnO nanoparticles, which weakened the proportion of C. Element O came from both ZnO and GO, and Zn represents the distribution of ZnO particles. EDX confirmed that ZnO nanoparticles were evenly dispersed on the surface of GO sheets. Next, X-ray diffractometry (XRD) was used to analyze the crystal of ZnO/GO nanocomposites (Figure 3a). We prepared pure ZnO for comparison and characterized GO and ZnO/GO (Figure 3b). The diffraction peak of GO at 2θ = 10.8° shows the (001) crystal planes of GO [21]. In ZnO/GO spectrum, the peaks appeared at 31.8°, 34.5°, 36.3°, 47,6°, 56,6°, 62.9°, 68.0° and 69.1° corresponding to (100), (002), (101), (102), (110), (103), (112) and (201) crystal planes of ZnO respectively (JCPDS no. 36-1415), and the no GO peak is found may be due to the content of GO was low and GO was covered by ZnO nanoparticles [22,23]. Figure 3b shows the spectra of ZnO/GO and GO. The three peaks that GO exhibited at 1737, 1620 and 1050 cm^−1^ can be assigned to the stretching vibration of oxygen-containing carboxyl (C=O), skeletal vibration of graphene sheets and alkoxy (C–O) functional group, respectively [24]. ZnO/GO exhibited not only a stretching vibration peak of Zn–O bond at 559 cm^−1^, but also the skeletal vibration of the graphene sheets at 1572 cm^−1^ and alkoxy (C–O) functional group at 1008 cm^−1^ [25]. The changes of these functional groups proved that GO could combine with ZnO effectively, and GO partially reduced during the hydrothermal process [26], thus decreasing the number of functional groups.

#### 2.1.2. Characterization of ZnO/GO-Gelatin Fibers

**Figure 4:**
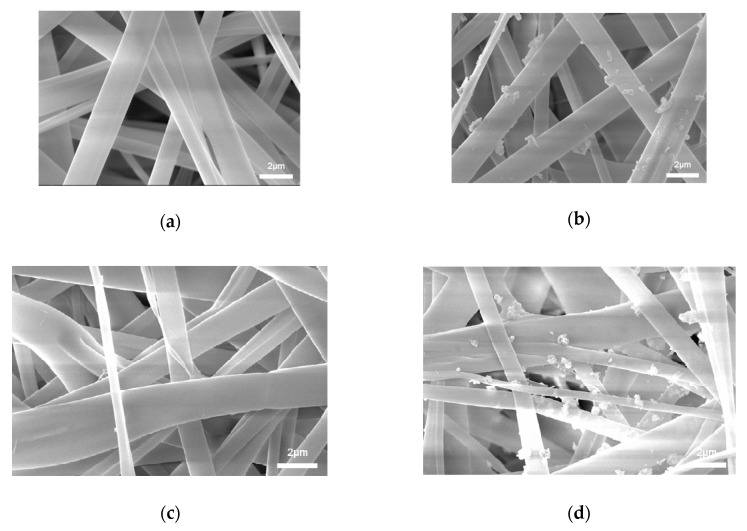
FESEM images of (**a**) GELF’, (**b**) ZGF’, (**c**) GELF and (**d**) ZGF.

**Figure 5:**
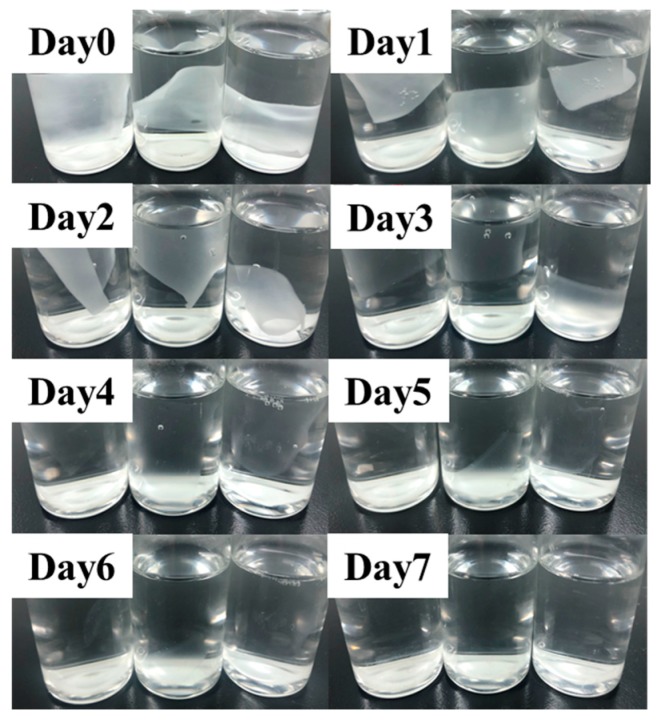
The degradation process of ZGF in phosphate buffer solution (PBS) at 37 °C.

FESEM images in Figure 4a–d show the fibers before and after crosslinking. GELF′ and ZGF′ represent the pure gelatin fibers and ZnO/GO-gelatin fibers before crosslinking. GELF and ZGF represent the pure gelatin fibers and ZnO/GO-gelatin fibers after crosslinking. The pure gelatin fibers were smooth and continuous, with an average diameter of 1.39 μm before crosslinking. After ZnO/GO nanocomposites were sprayed on the surface of the gelatin fibers, the framework of the fibers showed no significant changes except for the uniform dispersion of ZnO/GO. To achieve the controllable degradation of fibers, we used formaldehyde to crosslink GELF′ and ZGF′. Crosslinking did not change the fibers′ framework; only the surface became uneven, and overlapping parts fused together. Moreover, the degradation process of ZGF in PBS at 37 °C is shown in Figure 5. The result indicated that different from uncross-linked gelatin fibers, which dissolve in aqueous solutions instantly, GEL and ZGF could be degraded completely in 7 days. Chen et al. used the steam of formaldehyde/ethanol solution to crosslink the gelatin membranes, which was basically degraded after 5 days [27]. Eldin et al. added glutaraldehyde into gelatin and chitosan mixture to crosslink the membranes; the weight loss of the crosslinked membranes was about 42% after 6 days [28]. Controllable degradation time is beneficial for the application as a wound dressing.

**Figure 6:**
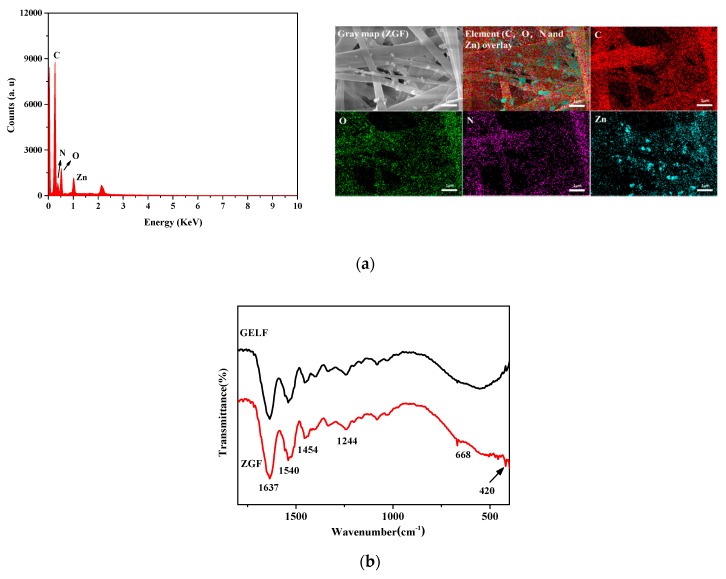
(**a**) EDX analysis of ZGF, (**b**) FTIR spectra of GELF and ZGF.

In order to determine the composition of the elements of ZGF, EDX and FTIR were carried out. As shown in Figure 6a, ZGF presented four elements mainly: C, N, O and Zn; gelatin contributed three elements: C, N and O mainly; and ZnO/GO nanocomposites contributed a small amount of C, O and all Zn elements. A new peak appeared at 420 cm^−1^ in the case of the fibers containing ZnO/GO corresponding to the stretching vibration peak of the Zn-O bond (Figure 6b), and an extra peak at 668 cm^−1^ could be assigned to the metal-oxygen stretching mode [16], which represents that ZnO/GO have been incorporated in the gelatin fibers successfully. The characteristic peaks of amid I, amid II and amid III observed at 1637, 1540 and 1244 cm^−1^, respectively, both in GELF and ZGF were mainly attributed to –C=O stretching, –NH stretching and –C–N stretching, respectively [27]. In addition, the characteristic peak at 1454 cm^−1^ was mainly attributed to the stretching vibration peak of the imide group (–CH=N), which was caused by the reaction between the aldehyde group of formaldehyde and the amino lysine residue of gelatin [29].

**Figure 7:**
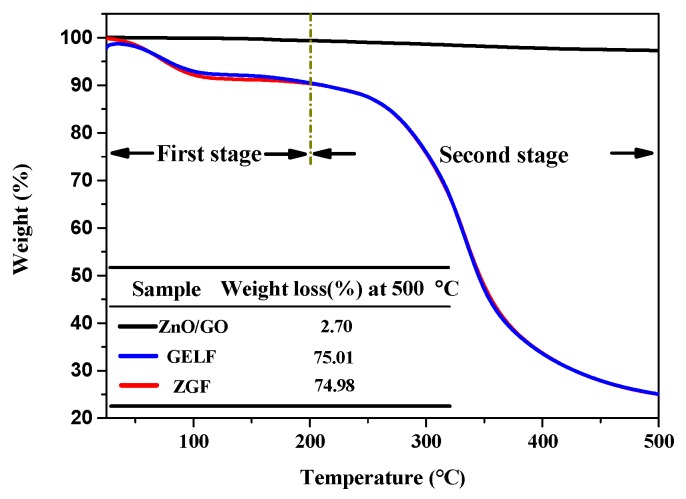
TGA curves of ZnO/GO, GELF and ZGF.

The thermal stability of ZGF was obtained by thermogravimetric analysis (TGA). As shown in Figure 7, the weight loss of ZnO/GO nanocomposites was 2.7% at 500 °C due to the evaporation of physically adsorbed water. GELF and ZGF had similar TGA curves and both could be divided into two stages [30,31]. The first stage between 25–200 °C, approximately 10% of weight lost, was caused by the evaporation of physically adsorbed water. The weight lost in the second stage could be assigned to the decomposition of gelatin, and ZGF showed less weight loss at 500 °C with the addition of ZnO/GO. TGA indicated that ZGF had a good thermal stability under 200 °C.

### 2.2. Antibacterial Assay

**Figure 8:**
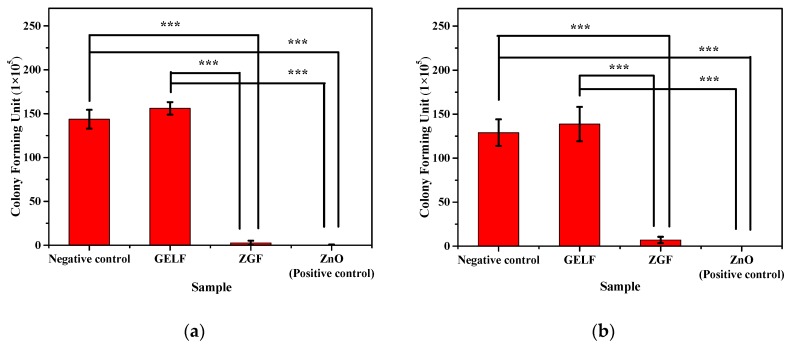
Antibacterial activities of negative control, GELF, ZGF and ZnO (positive control) to (**a**) *E. coli* and (**b**) *S. aureus*. (* represents *p* < 0.05, ** represents *p* < 0.01 and *** represents *p* < 0.001).

**Figure 9:**
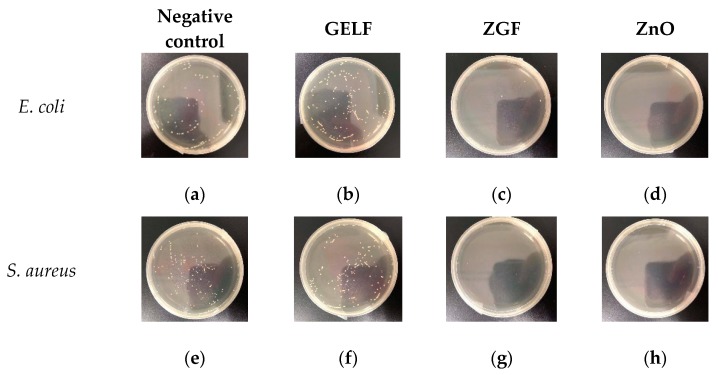
The photographs of antibacterial activities of negative control, GELF, ZGF and ZnO (positive control) to *E. coli* (**a**–**d**); to *S. aureus* (**e**–**h**).

Figure 8 illustrates the antibacterial activities of different samples against *E. coli* and *S. aureus* that were tested by the colony count method. Figure 9 represents photographs showing the antibacterial activities of ZGF. GELF showed no inhibition of bacterial growth [32], while ZGF indicated good antibacterial activities against *E. coli* and *S. aureus*, with the inhibition rates of 99.7% and 94.5%, respectively. The difference in the antibacterial activities of ZGF against the two bacteria may be attributed to the different cell wall structures of Gram-positive bacteria and Gram-negative bacteria [33]. According to the reported studies, the antibacterial mechanisms of the ZGF include not only the direct contact between ZnO nanoparticles and bacteria but the release of zinc ions and the production of reactive oxygen species (ROS) [13,34]. In addition, because of high absorption capacity for Zn^2+^, GO is also able to become a storage place for Zn^2+^ released from ZnO nanoparticles and increases the permeability of the cell membrane by contacting with negatively charged bacteria, which eventually lead to the deformation of the cell membrane and leakage of intracellular substances [35,36]. In addition, some researchers have proposed that ZnO, as a semiconductor, can generate electron–hole pairs and ROS under the irradiation of UV, which also leads to protein peroxidation, lipid peroxidation and DNA damage [36,37]. The unique two-dimensional sheet layer of GO will enhance the electron transfer rate and reduce the electron–hole pair recombination, which also makes ZnO/GO nanocomposites exert better antibacterial activities [38].

## 3. Materials and Methods

### 3.1. Materials

Gelatin (type B, basic-processed, prepared by bones, with a molecular weight of 100,000, viscosity value of 4.9 MPa·s^−1^) was obtained from Dongbao Bio-Tech Co., Ltd. (Baotou, China). 2,2,2-trifluoroethanol, formaldehyde solution (37–40 wt%), zinc acetate dihydrate (Zn(CH_3_COO)_2_·2H_2_O), diethylene glycol and ethanol absolute were purchased from Titan Scientific Co., Ltd. (Shanghai, China). GO powder was purchased from Tanfeng Graphene Technology Co., Ltd. (Suzhou, China). Phosphate buffer solution (PBS, powder, 0.01 M, pH = 7.2–7.4) was purchased from Solarbio Science & Technology Co., Ltd. (Beijing, China). Tryptone soy broth (TSB) was purchased from Aoboxing Biotechnology Co., Ltd. (Beijing, China). *E. coli* and *S. aureus* were supplied by the China General Microbiological Culture Collection Center (Beijing, China). All reagents were of analytical grade and were utilized as received.

### 3.2. Preparation of ZnO/GO Nanocomposites

For the preparation of ZnO/GO nanocomposites, 658 mg (0.003 mol) of zinc acetate dihydrate and GO powder (5 wt%, 10 wt% and 15 wt%, respectively) were put into a beaker containing 30 mL diethylene glycol with continued ultrasonic until dissolved completely. The mixture was then transferred to a Teflon-lined stainless steel autoclave (50 mL) and heated to 140 °C for 5 h. After reaction, the solutions were naturally cooled to room temperature. Finally, the product was obtained after ultrasonic, washing, and drying at 100 °C for 5 h. Labeling the products as ZnO/GO-5 wt%, ZnO/GO-10 wt% and ZnO/GO-15 wt% according to the different addition of GO.

### 3.3. Preparation of ZnO/GO-Gelatin

First, an amount of 30 mg GO was dispersed in 10 mL absolute ethanol by ultrasonic. Then gelatin (10%, *w/v*) was added into 2,2,2-trifluoroethanol, and stirred until completely dissolved at 50 °C. The two solutions were transferred to syringes (20 mL), the distance between the needle tip and the drum collector wrapped in aluminum foil was adjusted to 15 cm, the applied voltage was 17 kV, the drum collector rotation speed was 120 rpm, and the gelatin fibers and ZnO/GO nanocomposites were spun with a flow rate of 5 and 2.5 mL·h^−1^. In addition, pure gelatin fiber, without adding any materials, was prepared as a control. After electrospinning, fibers were put on the upper layer of the dryer with 1 mL formaldehyde in the lower layer for 12 h. Then, the fibers were taken out and ventilated in the fume hood for 48 h to remove the residual formaldehyde solution.

### 3.4. Characterization

The microstructure and elements distribution were characterized by FESEM (QUANTA FEG 250, FEI, Hillsboro, OR, USA) and EDX (QUANTA FEG 250, FEI, Hillsboro, OR, USA). ZnO diameter distribution was determined using Nano Measurer 1.2 software by measuring the diameter of 50 random ZnO nanoparticles. The functional groups in the nanoparticles and fibers were characterized by FTIR (Excalibur 3100, Varian, Palo Alto, CA, USA), and the scanning range of the samples was 400–4000 cm^−1^. The crystal structures of the samples were determined by XRD (D8 focus, Bruker, Karlsruhe, Germany). The scanning rate was 0.1 s·step^−1^ and the scanning range was 5–70°. The thermal stability of the samples was analyzed by TGA (STA449C, NETZSCH, Selb, Germany) in a nitrogen atmosphere, and the heating rate was 10 °C·min^−1^.

### 3.5. Stability of ZGF in PBS

In order to test the stability of the crosslinked fibers, GELF and ZGF were cut into small pieces of 2 × 3 cm^2^ and put into a bottle containing 15 mL PBS. Then, the bottles were put into a 37 °C oven and recorded every 24 h. The experiment was performed in triplicate.

### 3.6. Antibacterial Assay

The antimicrobial activities of ZGF against *E. coli* and *S. aureus* were determined by the colony count method. First, the sterile GELF and ZGF were cut into small pieces of 3 × 4 cm^2^, and then they were irradiated for 1h under an ultraviolet lamp (UV, 365 nm, 40 W) after being put into tubes containing 10 mL of sterile PBS. The bacteria PBS suspensions were added into the tubes containing GELF and ZGF, respectively, and put into a shaker at 37 °C. The bacteria PBS suspensions were taken out after 3 h. The bacterial solution was diluted by gradient and spread on tryptone soybean agar (TSA) for 14 h at 37 °C. The number of colonies formed were counted. In addition, the blank bacterial solution was used as the negative control, and the bacterial solution of ZnO (30 mg) particles were used as the positive control. All the experiments were in triplicate.

### 3.7. Statistical Analysis

The data were expressed as mean ± standard deviation. Statistically significant differences in the samples were assessed using IBM SPSS Statistics 20.0. (International Business Machines Corporation, Armonk, NY, USA) *p* < 0.05 was considered to be statistically significant.

## 4. Conclusions

In summary, it is the first time that ZnO/GO nanocomposites were incorporated into gelatin fibers by side-by-side electrospinning technique to serve as a wound dressing. The characterization showed that the ZnO/GO nanocomposites were uniformly dispersed on the fibers with good morphology. After crosslinking, the fibers have controllable degradation time and could be completely degraded within 7 days. The antibacterial assay indicated that the antibacterial rate of the composite fiber material to *E. coli* and *S. aureus* was more than 90%. The experimental results suggest that the gelatin fibers containing ZnO/GO with controllable degradation time and excellent antibacterial performance have a promising prospect as wound dressing material.
